# Positive hepatitis B surface antigen tests due to recent vaccination: a persistent problem

**DOI:** 10.1186/1472-6890-12-15

**Published:** 2012-09-24

**Authors:** Carolyn D Rysgaard, Cory S Morris, Denny Drees, Tami Bebber, Scott R Davis, Jeff Kulhavy, Matthew D Krasowski

**Affiliations:** 1 Department of Pathology, University of Iowa Hospitals and Clinics, Iowa City, IA, 52242, USA

**Keywords:** False positive reactions, Hepatitis, Hepatitis B surface antigens, Public health, Renal dialysis, Vaccination

## Abstract

**Background:**

Hepatitis B virus (HBV) is a common cause of viral hepatitis with significant health complications including cirrhosis and hepatocellular carcinoma. Assays for hepatitis B surface antigen (HBsAg) are the most frequently used tests to detect HBV infection. Vaccination for HBV can produce transiently detectable levels of HBsAg in patients. However, the time course and duration of this effect is unclear. The objective of this retrospective study was to clarify the frequency and duration of transient HBsAg positivity following vaccination against HBV.

**Methods:**

The electronic medical record at an academic tertiary care medical center was searched to identify all orders for HBsAg within a 17 month time period. Detailed chart review was performed to identify all patients who were administered HBV vaccine within 180 days prior to HBsAg testing and also to ascertain likely cause of weakly positive (grayzone) results.

**Results:**

During the 17 month study period, 11,719 HBsAg tests were ordered on 9,930 patients. There were 34 tests performed on 34 patients who received HBV vaccine 14 days or less prior to HBsAg testing. Of these 34 patients, 11 had grayzone results for HBsAg that could be attributed to recent vaccination. Ten of the 11 patients were renal dialysis patients who were receiving HBsAg testing as part of routine and ongoing monitoring. Beyond 14 days, there were no reactive or grayzone HBsAg tests that could be attributed to recent HBV vaccination. HBsAg results reached a peak COI two to three days following vaccination before decaying. Further analysis of all the grayzone results within the 17 month study period (43 results out of 11,719 tests) revealed that only 4 of 43 were the result of true HBV infection as verified by confirmatory testing.

**Conclusions:**

Our study confirms that transient HBsAg positivity can occur in patients following HBV vaccination. The results suggest this positivity is unlikely to persist beyond 14 days post-vaccination. Our study also demonstrates that weakly positive HBsAg results often do not reflect actual HBV infection, underscoring the importance of confirmatory testing. This study also emphasizes that vaccination-induced HBsAg positives occur most commonly in hemodialysis patients.

## Background

Hepatitis B virus (HBV) remains a common cause of viral hepatitis, with possible long-term complications of cirrhosis and hepatocellular carcinoma in patients who develop chronic infection [1,2]. A variety of serological and molecular-based laboratory assays are used to diagnose and manage HBV infection [2]. Hepatitis B surface antigen (HBsAg) remains one of the most frequently ordered tests. In both acute and chronic HBV infection, HBsAg can be detected. HBsAg testing is commonly used in the hemodialysis population to detect HBV infection [3]. Patients who are documented as HBV-positive utilize dedicated work areas and equipment to avoid spread of HBV to other dialysis patients.

Vaccination to HBV has had a significant impact on reducing infection [1,2]. However, one consequence of HBV vaccination is the possibility of positive HBsAg tests since HBV vaccine contains surface antigen [4,5]. In cases of recent vaccination, a positive HBsAg test can lead to unnecessary confirmatory laboratory testing, with added expense for the patient as well as the stress of a false positive result. Unrecognized false positives can lead to additional downstream effects such as reporting to public health authorities and use of dedicated dialysis equipment for renal failure patients. Positive HBsAg results following HBV vaccination have been reported in a variety of patient populations: neonates [6-10], hemodialysis patients [4,11-13], blood donors [5,14,15], and general adult patients [16-18]. The present study aims to clarify the nature and duration of transient post-vaccination positivity and how common vaccine-induced positive results occur in an academic center population.

## Methods

### Setting

We conducted a retrospective analysis of electronic laboratory and medical records from a tertiary care academic medical center. The project had Institutional Review Board approval from the University of Iowa. All research carried out in human subjects was in compliance with the Helsinki Declaration.

### Selection of study subjects

The HBsAg testing used in this study was the cobas® hepatitis B surface antigen assay (Roche Diagnostics, Indianapolis, IN, USA). The electronic medical record (Epic, Epic Systems Inc., Madison, WI, USA) was searched for the time period from April 2010 to September 2011 for patients who had HBsAg testing performed. The inclusion criteria was having an HBsAg test ordered and resulted. Instances where HBsAg testing was ordered but unable to be performed due to interference (e.g., hemolyzed specimen) or inadequate specimen were excluded. By institutional policy for the hospital core clinical laboratory, a reactive result for HBsAg was defined by a Cut-Off Index (COI) of > 20. A weakly reactive (“grayzone”) result was defined by COI ≥ 1 to ≤ 20. All reactive and grayzone results were automatically re-tested, with persistent grayzone results sent to a reference laboratory (ARUP Laboratories, Salt Lake City, UT, USA) for confirmatory testing by HBsAg neutralization assay. Chart review, including review of pharmacy records, was performed for patients with grayzone and reactive results. Two HBV vaccines were used during the period of this retrospective study: Engerix-B® (at either 20 mcg or 40 mcg dose, GlaxoSmithKline, Brentford, Middlesex, United Kingdom) and a combined hepatitis A/hepatitis B vaccine (Twinrix®, GlaxoSmithKline). Administration of these vaccines in both the inpatient and outpatient setting was documented within the electronic medical record medicine administration record.

### Data analysis

Statistical analyses were carried out in EP Evaluator release 9 (Data Innovations, South Burlington, VT, USA).

## Results

### HBsAg positivity due to recent vaccination

**Figure 1:**
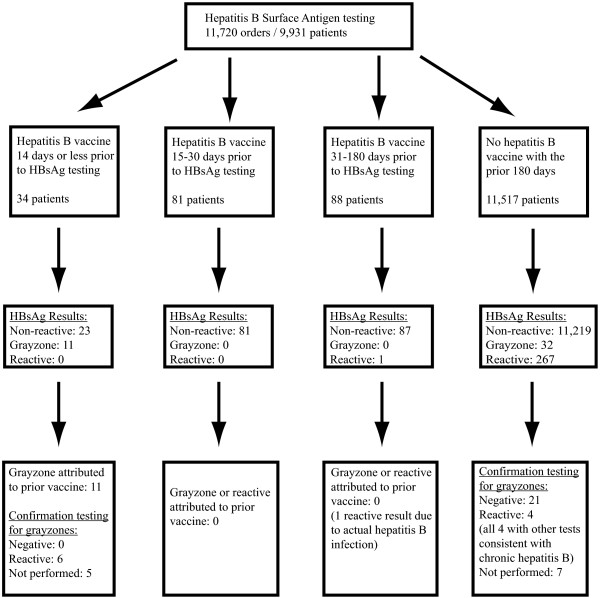
Flow diagram illustrating the derivation of the study sample.

In the seventeen month time period of this retrospective study, there were 11,719 HBsAg tests ordered for 9,930 patients (with some patients being tested two or more separate times during the time period of the study), with a total of 43 grayzone and 268 reactive results (see Figure 1). Thirty-four HBsAg tests were performed on 34 different patients within 14 days of a dose of Engerix-B® hepatitis B vaccine, with 24 of these occurrences involving patients with chronic renal failure who were tested prior to a hemodialysis treatment (Additional File 1). Of these 34 tests, 11 were resulted as grayzone; the remaining 23 were reported as non-reactive (COI < 1). Patients with grayzone results received the vaccine at a dose of either 20 mcg (1 patient) or 40 mcg (10 patients). Demographic, medical history, other laboratory testing, and nutritional status of the 34 patients at the time of HBsAg testing is detailed in Additional File 1.

In 10 of the 11 patients, the hepatitis B vaccine was ordered by a different provider from the provider who ordered the HBsAg test. These 10 patients were all patients undergoing routine HBsAg surveillance testing in the setting of receiving chronic hemodialysis for renal failure, with all seven hemodialysis patients who had HBsAg testing 5 days or less following a vaccine dose having a grayzone reaction. One of the 11 patients had hepatitis B vaccine and HBsAg testing both done as part of the same outpatient encounter, with the vaccine administered approximately 6 hours prior to the blood draw for HBsAg testing. This patient received HBsAg testing as part of medical testing prior to travel to another country. For all 11 patients, there was no clinical suspicion of active viral hepatitis; HBsAg testing was done for screening purposes. Nine of the 12 patients administered hepatitis B vaccine 6 days or less prior to HBsAg testing had a grayzone reaction; in contrast, only 2 of 22 patients who were tested 7 to 14 days after a vaccine dose had a grayzone reaction (Additional File 1). The longest interval between vaccination and grayzone reaction was 12 days. All 11 patients with vaccine-induced grayzone results had subsequent HBsAg performed anywhere from 7 to 28 days later (and in many cases multiple times thereafter); in each case, follow-up testing was negative (Additional File 1). In addition, 9 of the 11 patients had had prior HBsAg testing done anywhere from 12 days to 6 months prior to vaccination, with negative HBsAg results in each case (Additional File 1). None of the vaccine-induced HBsAg grayzone reactions occurred in the pediatric population, despite children under 18 years old comprising 28.1% of the total HBsAg testing volume.

**Figure 2:**
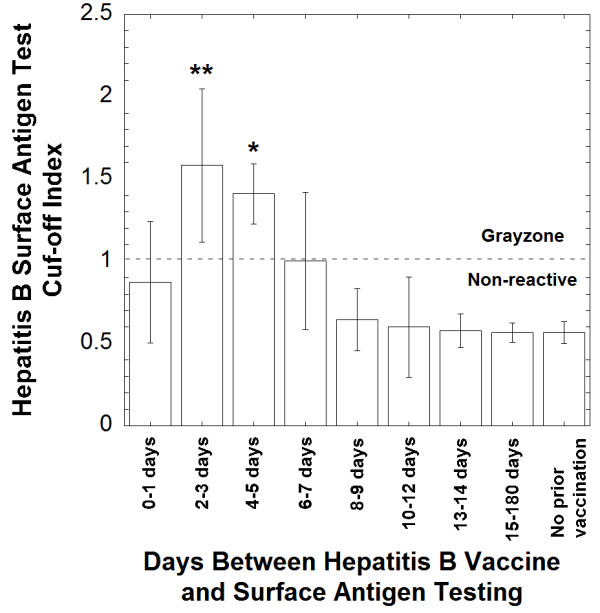
**Cut-Off Index for the hepatitis B surface antigen test for patients who received prior hepatitis B vaccination.** The Cut-Off Index (COI) for patients receiving vaccine 2–3 days and 4–5 days post-vaccine was statistically significant in comparison to the COI of non-reactive hepatitis B surface antigen testing in patients who did not receive prior vaccination (unpaired t-tests with p < 0.01, indicated by **, and p < 0.05, indicated by *, respectively).

**Figure 3:**
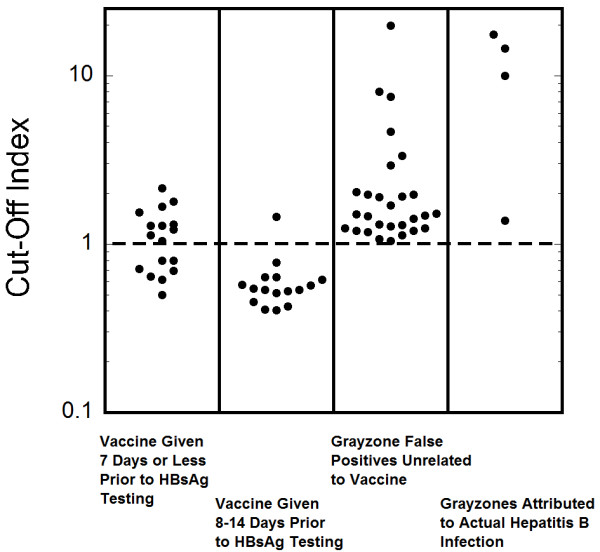
**Dot plots of Cut-Off Index.** The Cut-Off Index is plotted for four populations: patients who received hepatitis B vaccine 7 days or less prior to HBsAg testing, patients who received vaccine 8–14 days prior to HBsAg testing, patients with grayzone HBsAg reactions not attributed to vaccine or to actual hepatitis B virus infection, and patients with grayzone reactions who appear to have actual hepatitis B virus infection. The dashed line is at the Cut-Off Index of 1 which divides non-reactive from grayzone.

Further analysis showed the COI for the HBsAg assay reached a peak of 1.583 ± 0.464 at two to three days following vaccine administration before decaying to an average of 0.578 ± 0.102 by day 14. After 5 days post-vaccination, the COI was overall statistically not different from the average COI of patients non-reactive for HBsAg who had no prior vaccination (Figure 2). A dot plot showing COI for the HBsAg test for all 34 patients who received hepatitis B vaccine 14 days or less before HBsAg testing is shown in Figure 3. There was one patient who had a positive HBsAg result 78 days after vaccination with Engerix-B® vaccine. However, this patient had a documented history of chronic HBV infection and was further found to have 49,000 IU/mL of hepatitis B DNA on follow-up testing performed by a reference laboratory, demonstrating active HBV infection. With the exception of this single patient, no reactive or grayzone results were reported in patients who were tested for HBsAg between 15 and 180 days following a Engerix-B® vaccination dose (167 patients) or for two patients who received a dose of the Twinrix® combined hepatitis A/hepatitis vaccine within 14 days of HBsAg testing.

### Other causes of grayzone results

There were 32 patients with grayzone results for HBsAg who had no history of HBV vaccine administration within the prior 180 days. Demographic, medical history, other laboratory testing, and nutritional status of these 32 patients at the time of HBsAg testing is detailed in Additional File 2. Of these, 21 had a negative result by confirmatory neutralization assay and can thus be definitively considered false positives. These 21 patients were from a fairly diverse patient population including pregnant women in first or second trimester (n = 7) and patients with rheumatoid arthritis (n = 2), new onset heart failure (n = 1), ulcerative colitis (n = 1), and pulmonary hypertension (n = 1). Unlike the population of patients who had suspected hepatitis B vaccine-induced HBsAg grayzone results discussed above, only 2 of the 32 patients had chronic renal failure. Four patients with grayzone results were verified by other laboratory testing to have chronic HBV infection, which was also consistent with clinical history and physical examination (Additional File 2). Seven patients, including a one month old infant with cholestatic jaundice, had a grayzone HBsAg result but no neutralization assay follow-up; one of these patients (25 year old male) was negative on a repeat HBsAg test performed 5 days later. Chart review revealed physician documentation of low clinical suspicion of hepatitis B in all seven patients. Thus, out of the total of 32 patients with grayzone results, only 4 had clinical history and laboratory testing consistent with actual HBV infection (Additional File 2). The COI for the HBsAg for the 4 patients with confirmed chronic HBV infection and a grayzone reaction was 10.8 ± 7.0. The COI for the remaining 28 patients with grayzone HBsAg results was 2.8 ± 3.8. A dot plot showing COI for the HBsAg test for all 32 patients with grayzone reactions not associated with recent hepatitis B vaccination is shown in Figure 3. For comparison, the average COI for the 267 reactive HBsAg results was 3221 ± 706.

## Discussion

The results of this study confirm that transient HBsAg positivity can occur in patients who have recent HBV vaccination. In our academic medical center population, we found 11 cases out of 11,719 total tests (0.085%) where vaccination caused weakly positive (grayzone) HBsAg results. These 11 cases arose out of 34 instances where HBV vaccine was administered within 14 days of HBsAg testing. Ten of the 11 cases involved the higher (40 mcg) of the two doses available for the Engerix-B® vaccine.

In all 11 patients, HBsAg testing was for screening purposes (either for dialysis patients or pre-travel laboratory tests) and not for work-up of clinically suspected viral hepatitis infection. In addition, except for one patient who received vaccination and HBsAg testing in the same outpatient visit, the other ten patients were all hemodialysis patients who had the HBsAg test ordered as part of ongoing surveillance and the HBV vaccine administered during an outpatient visit to a different provider (usually a primary care physician or registered nurse practitioner). Our study is consistent with other studies demonstrating hepatitis B vaccine-induced reactive HBsAg test in hemodialysis patients [4,11-13].

This study highlights why vaccine-induced positivity for the HBsAg test is a persistent problem that is difficult to eliminate. One would predict that the challenge is even greater if a patient is seen in two different healthcare systems that do not share a common electronic medical record. In our own medical center, we have now instituted a “Best Practice Alert” in our electronic medical record that alerts the physician when a HBsAg test is ordered within 14 days of a HBV vaccine order and advises delaying HBsAg testing until at least 15 days after vaccination.

The hemodialysis population is particularly challenging with regard to HBsAg testing as patients often undergo regular HBsAg testing and may be seen by other providers for a variety of other medical issues. In addition, hemodialysis patients tend to respond poorly to hepatitis B vaccines and thus frequently receive a higher dose of vaccine than used in other patient populations. Hemodialysis patients may even require additional doses of vaccine to achieve adequate levels of antibodies against HBsAg [11].

There are two main limitations to the analysis presented in this paper. First, although pediatric patients 18 years old or less comprised 28.1% of the total HBsAg tests, there were no pediatric patients that received hepatitis B vaccine within 14 days of HBsAg testing. Consequently, the results related to recent vaccine administration are only applicable to adult patients. However, the sample size of this study exceeds that of previous studies and contains a patient population that likely is similar to that analyzed by clinical laboratories at many academic medical centers, and has produced results comparable to other similar studies of academic medical center patient populations [4,11-13]. Second, although nutritional status was not examined in detail, it is likely that most patients in the study were well-nourished and thus the findings are most applicable to other well-nourished populations.

Our study also demonstrates that weakly positive HBsAg results uncommonly reflect actual HBV infection. Of the overall 43 grayzone results out of 11,719 total tests, only 4 patients were found to have active HBV infection by additional confirmatory laboratory work-up, e.g., HBV DNA although it should be pointed out that not all patients had detailed confirmatory work-up performed. Reactive (not grayzone) HBsAg tests were often markedly higher than the upper limit of the grayzone (COI = 20) and generally had COIs of greater than 1,000. We believe our results should prompt caution in interpretation of weakly positive HBsAg results, at least for the Roche Diagnostics assay used, and also highlights the importance of follow-up confirmatory testing to ascertain true infection status and avoid the downstream consequences of false positives.

## Conclusions

Our study confirms that transient HBsAg positivity can occur in patients following HBV vaccination, usually not extending beyond 14 days post-vaccination. This study also emphasizes that renal dialysis patients are a population that vaccination-induced HBsAg positives most commonly occurs. Decision support algorithms may be effective in preventing attempts to order HBsAg testing too soon following recent HBV vaccination.

## Competing interests

The authors all declare no competing interests.

## Authors’ contributions

CDR and MDK were involved in the study concept and design, analysis and interpretation of the data, drafting and revisions of the manuscript. CSM, DD, TB, SRD, and JK contributed to data analysis and critical interpretation of the laboratory testing. All authors have read and approval the final manuscript.

## Pre-publication history

The pre-publication history for this paper can be accessed here:

http://www.biomedcentral.com/1472-6890/12/15/prepub

## Supplementary Material

Additional file 1 Demographics, laboratory studies, and medical histories of patients with HBsAg testing within 14 days of hepatitis B vaccination. Click here for file

Additional file 2 Demographics, laboratory studies, and medical histories of patients with grayzone HBsAg results not attributed to recent vaccination. Click here for file
